# Expression of the SARS-CoV-2 receptor ACE2 reveals the susceptibility of COVID-19 in non-small cell lung cancer

**DOI:** 10.7150/jca.49462

**Published:** 2020-07-09

**Authors:** Hongming Zhang, Kelly Quek, Runzhe Chen, Jibei Chen, Baoan Chen

**Affiliations:** 1 Department of Respiratory Medicine, Yancheng Third People's Hospital, the Affiliated Yancheng Hospital of Southeast University Medical College, Yancheng, Jiangsu Province, P.R. China; 2 Thoracic/Head & Neck Medical Oncology, University of Texas MD Anderson Cancer Center, Houston, Texas, USA; 3 Department of Hematology and Oncology, Zhongda Hospital, Medical School, Southeast University, Jiangsu Province, P.R. China

**Keywords:** ACE2, receptor, expression, COVID-19, SARS-CoV-2, survival

## Abstract

Recent studies have revealed that cancer patients had a higher risk of having coronavirus disease 2019 (COVID-19), caused by severe acute respiratory syndrome coronavirus 2 (SARS-CoV-2), compared to patients without cancer. The expression of angiotensin-converting enzyme 2 (ACE2), the receptor of SARS-CoV-2, was aberrantly expressed in many tumors. In this study, by exploring the TCGA and GTEx public databases, we investigated ACE2 expression and its association with prognosis in non-small cell lung cancer (NSCLC), the most susceptible caner type. We found that lung was one of the major organs with highly expressed ACE2. Furthermore, ACE2 expression was significantly elevated in lung adenocarcioma (LUAD) and lung squamous cell carcinoma (LUSC) compared to normal tissues. DNA methylation might be one possible mechanism leading to ACE2 upregulation. Despite that, the AEC2 expression was not statistically associated with disease-free survival (DFS) and overall survival (OS) for LUAD patients, and higher ACE2 expression was associated with prolonged DFS in LUSC patients. Taken together, we observed ACE2 was highly expressed in LUAD and LUSC despite the controversial role of ACE2 expression in predicting prognosis in these two common lung cancer types.

## Introduction

Since the first case of a novel coronavirus disease 2019 (COVID-19) caused by severe acute respiratory syndrome coronavirus 2 (SARS-CoV-2) in early December 2019, it has spread rapidly all over the world and been considered as a global public health concern 1. As of May 28 2020, more than 5.6 million of confirmed cases with 353 thousand deaths have been reported globally 2. Considering that cancer patients are regarded as a highly vulnerable group in the current COVID-19 pandemic, especially in patients with lung cancer 3, 4, it is important to investigate the susceptibility of lung cancer to SARS-CoV-2 molecularly. As the expression of angiotensin-converting enzyme 2 (ACE2), the receptor of SARS-CoV-2, was aberrantly expressed in many tumors, in this study we investigated expression of ACE2 5, 6 in non-small cell lung cancer (NSCLC) including lung adenocarcinoma (LUAD) and lung squamous cell carcinoma (LUSC) and its association with prognosis with the aim of understanding the role of ACE2 expression in lung cancer survival.

## Methods

Gene expression data of tumor and normal samples of lung cancer patients from The Cancer Genome Atlas (TCGA) and Genotype-Tissue Expression (GTEx) databases were acquired from Gene expression profiling interactive analysis 2 (GEPIA2) (http://gepia2.cancer-pku.cn) tool 7. Survival analysis was also performed on GEPIA2. Analysis of DNA methylation level of ACE2 promoter in lung cancers from TCGA were conducted by UALCAN (http://ualcan.path.uab.edu/) tool 8.

## Results

**Figure 1:**
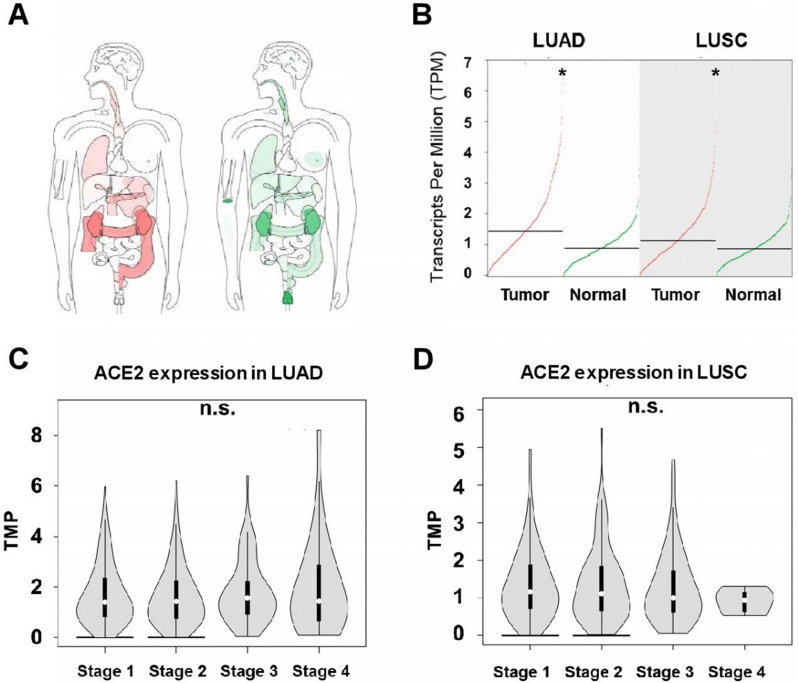
** Expression of angiotensin-converting enzyme 2 (ACE2) in non-small cell lung cancer (NSCLC). A)** The median ACE2 expression of tumor (red) and normal (green) samples in bodymap (Log_2_ (TPM + 1) Scale, the color depth indicates the degree of ACE2 expression). **B)** Summary of ACE2 expression in tumor and normal tissues of lung adenocarcinoma (LUAD) and lung squamous cell carcinoma (LUSC). ACE2 expression in different pathological stages of **C)** LUAD and **D)** LUSC.

We first carried out a pan-caner analysis of ACE2 expression in tumor and its normal control tissues cross the whole body of human beings from TCGA and GTEx databases. As shown in **Figure 1A**, lung is one of the major organs with ACE2 highly expressed. In the two types of NSCLCs, expression of ACE2 was significantly upregulated in tumors compared to normal tissues, with a median level of 1.44 in tumor and 0.89 in normal tissue in LUAD (482 tumors *versus* 347 normal, p<0.05), and a median level of 1.13 in tumor and 0.87 in normal tissue in LUSC (486 tumors *versus* 338 normal, p<0.05) (**Figure 1B**). Interestingly, when looking at ACE2 expression in different pathological stages (1 to 4), no differences was observed in any of the two lung cancer types (**Figure 1C-D**), suggesting stage might not the factor affecting ACE2 expression in lung tumor and therefore no significant differences in the susceptibility to SARS-CoV-2 infection among the pathological stages for LUAD and LUSC patients.

**Figure 2:**
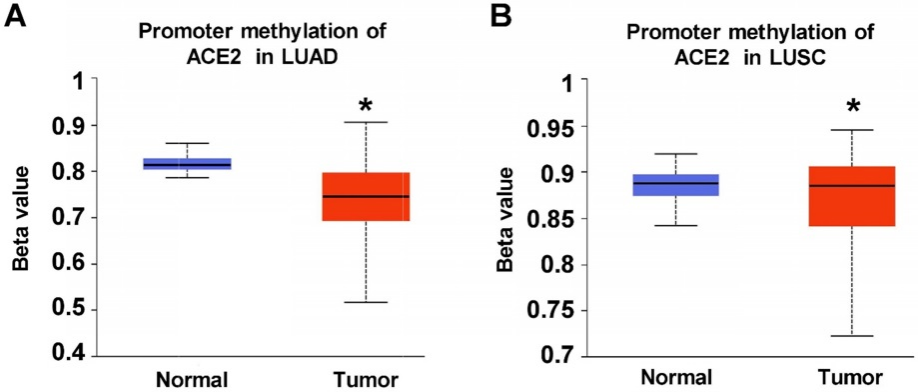
**DNA methylation level of ACE2 promoter in TCGA non-small cell lung cancer (NSCLC).** Promoter methylation of ACE2 in **A)** LUAD and **B)** LUSC.

Considering that DNA hypermethylation be a potential mechanism of transcriptomic ACE2 abnormality in lung tumors 9, we next analyzed DNA methylation level of ACE2 promoter in TCGA lung cancers. As shown in Figure **2A-B**, with beta value ranging from 0 (unmethylated) to 1 (fully methylated), significantly decreased DNA methylation level of ACE2 was detected in both LUAD and LUSC tumors compared with their paired normal lung tissues (LUAD: 473 tumors *versus* 32 normal, p<0.05; LUSC: 370 tumors *versus* 42 normal). This result suggests that DNA methylation might be one possible mechanism leading to upregulation of ACE2 expression in lung tumors.

**Figure 3:**
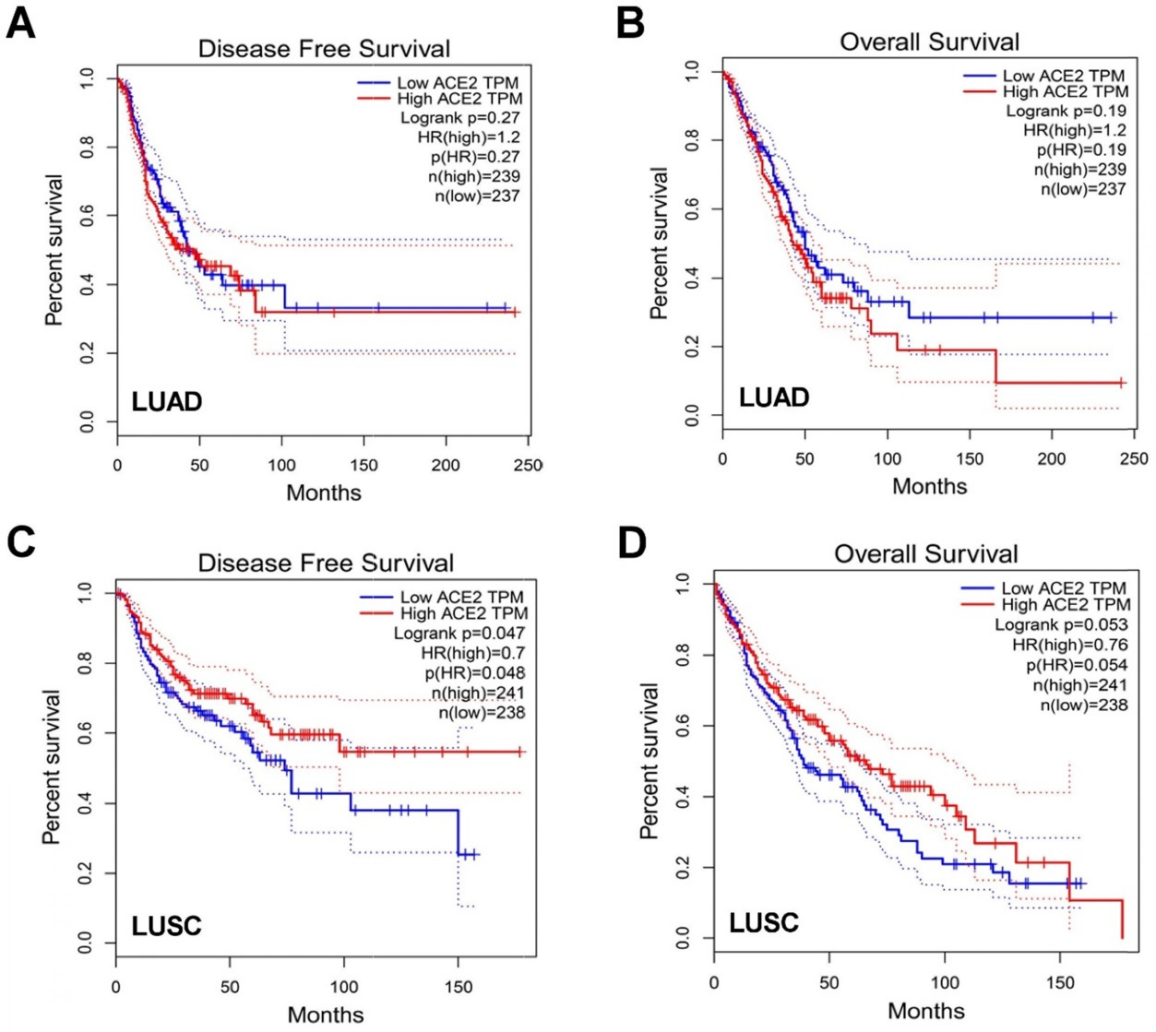
**Association of ACE2 expression with clinical outcome.** Impact of ACE2 expression on **A)** disease-free survival (DFS) and **B)** overall survival (OS) in LUAD patients. Impact of ACE2 expression on **C)** DFS and **D)** OS in LUSC.

To further understand the impact of ACE2 in patient prognosis, we then explored differential expression of ACE2 in disease-free survival (DFS) and overall survival (OS) among LUAD and LUSC patients. In LUAD patients, AEC2 expression was not statistically relevant to DFS or OS, however elevated ACE2 expression had a trend of worse OS (**Figure 3A-B**). Conversely, in LUSC patients, higher ACE2 expression was correlated with prolonged DFS (p=0.048) while no statistically difference for OS (**Figure 3C-D**). The different trends of ACE2 expression in LUAD and LUSC might indicate the dual-edged sword of ACE2 for patients' outcomes.

## Discussion

As the only experimentally confirmed SARS-CoV-2 receptor, ACE2 could facilitate virus for cellular entry and its expression level is considered to indicate the susceptibility of COVID-19 5, 6. In this study, we found elevated ACE2 expression in both lung tumors and normal tissues, which may explain the more inclination of SARS-CoV-2 infection in the respiratory system. ACE2 expression was higher in LUAD and LUSC compared to normal tissues and didn't vary in terms of stages, and this may show the susceptibility to SARS-CoV-2 among lung cancers regardless of stage. We also discovered DNA methylation aberration of ACE2 in lung tumors, and this might be one underlying mechanism leading to increased ACE2 expression. Other possibilities, such as histone modifications and glycosylation may also give rise in the abnormal expression of ACE2 10, 11, which requires further exploration. Furthermore, ACE2 has been proven to be an important regulator in tumorigenesis 12, 13. Patients with lung cancer were observed to harbor a higher incidence of COVID-19, with more severe symptoms 3, 4, 14. However, in this study, elevated ACE2 expression was numerically relevant to shorten OS in LUAD while significantly associated with better DFS in LUSC, suggesting a very complex relationship between ACE2 and lung cancer and the controversial role of ACE2 expression. Nevertheless, our findings may help gain more insight into the COVID-19 pathogenesis and design therapeutic strategies. Since our study is only a database analysis, further functional studies and validation in larger clinical cohort is warranted.

## Author Contributions

Study design: BC; Data collection and interpretation: HM, RC, BC; Original draft preparation: RC, HZ; Review and editing: RC, KQ, BC; Supervision: BC, RC, JC. All authors reviewed and approved the final version of the manuscript.
